# Analysis of factors influencing the complications of severe acute pancreatitis combined with hemorrhage

**DOI:** 10.1186/s12876-026-05154-0

**Published:** 2026-07-27

**Authors:** Liang Shao, Jianhua Wan, Wenhua He, Yong Zhu, Yin Zhu, Jing Liu, Fen Liu, Nonghua Lu, Liang Xia

**Affiliations:** 1 Department of Gastroenterology, the 1st affiliated hospital, Jiangxi Medical College, Nanchang University, 17 Yongwaizheng Street, Nanchang, 330006 Jiangxi China; 2 Department of Pharmacy, the 1st affiliated hospital, Jiangxi Medical College, Nanchang University, Nanchang, China; 3 Department of Intensive Care Unit, the 1st affiliated hospital, Jiangxi Medical College, Nanchang University, Nanchang, China

**Keywords:** Severe acute pancreatitis, Hemorrhage complication, Influencing factors, Prognosis

## Abstract

**Background:**

Complications of hemorrhage in patients with severe acute pancreatitis (SAP) significantly impact prognosis. However, current research on this topic remains limited. This study aims to analyze the factors influencing hemorrhage in SAP patients.

**Methods:**

This study retrospectively analyzed clinical data from SAP patients admitted to our hospital between January 2019 and December 2022. Using propensity score matching and multivariate logistic regression analysis, we identified independent factors influencing hemorrhage. Additionally, we developed a nomogram prediction model for bleeding in SAP patients and generated an ROC curve to assess its accuracy. Prognosis was also evaluated.

**Results:**

Based on the exclusion criteria, 804 patients were included in the study, with 97 (12.1%) experiencing hemorrhagic complications. After applying propensity score matching, 440 patients were selected, including 88 with bleeding. Multivariate logistic regression analysis identified several independent factors influencing hemorrhage in SAP: computed tomography severity index (CTSI) score (OR: 1.771, 95% CI: 1.434–2.187, *P* < 0.001), infected pancreatic necrosis (IPN) (OR: 4.407, 95% CI: 1.854–10.476, *P* < 0.001), acute physiology and chronic health evaluation II (APACHE II) score ≥ 15 (OR: 12.323, 95% CI: 5.015–30.282, *P* < 0.001), abdominal compartment syndrome (ACS) (OR: 3.685, 95% CI: 1.462–9.287, *P* = 0.006), and continuous renal replacement therapy (CRRT) (OR: 0.251, 95% CI: 0.094–0.670, *P* = 0.006). A nomogram prediction model was constructed, achieving an area under the ROC curve (AUC) of 0.924. The median ICU stay and total hospital stay were significantly longer in the bleeding group compared to the non-bleeding group (30 vs. 7 and 48 vs. 18, *P* < 0.001). Additionally, the mortality rate was significantly higher in the bleeding group compared to the non-bleeding group (46.6% vs. 6.5%, *P* < 0.001).

**Conclusion:**

The occurrence of bleeding complications in SAP signifies a poor prognosis. Therefore, for patients with elevated CTSI and APACHE II scores, especially those with IPN and ACS, it is crucial to enhance monitoring for bleeding. If necessary, CRRT may be implemented to improve the condition and reduce the risk of hemorrhage.

**Supplementary Information:**

The online version contains supplementary material available at https://doi.org/10.1186/s12876-026-05154-0.

## Background

Acute pancreatitis (AP) is a prevalent acute disease of the digestive system, with an increasing incidence in recent years [1]. Approximately 20% of AP patients progress to SAP, which can lead to various complications such as infection, obstruction, and bleeding [2]. Although bleeding complications in SAP patients are rare, they significantly exacerbate the condition, complicate treatment, and worsen prognosis, often resulting in a high mortality rate [3]. Bleeding in SAP is typically classified into intra-abdominal bleeding and gastrointestinal bleeding based on its location and clinical manifestations, often occurring in the later stages of the disease [4]. Symptoms range from nonspecific abdominal pain to hypovolemic shock. Diagnosis primarily relies on clinical manifestations and imaging examinations [5]. The exact mechanism of hemorrhage caused by pancreatitis remains not fully understood. Factors such as pancreatic juice leakage, abnormal activation of digestive enzymes, inflammatory response, coagulation dysfunction, and vascular endothelial cell injury are thought to contribute to damage and bleeding in the pancreas and peripancreatic vessels [6]. Additionally, sustained pressure in arterial blood vessels can result in the formation and eventual rupture of pseudoaneurysms, causing bleeding. The inflammatory response may also induce thrombosis in adjacent veins, such as the splenic vein, leading to venous obstruction and portal hypertension, which further exacerbates bleeding. Overall, bleeding in severe acute pancreatitis may originate from vessel wall erosion, pseudoaneurysm rupture, abnormal coagulation function, pancreatic-derived portal hypertension, and invasive procedures like debridement [7, 8]. Despite advances in organ support therapy and intensive care improving the survival rates of SAP patients [9], these patients remain susceptible to systemic and local complications later in the disease course. This susceptibility explains why the reduction in early mortality has not significantly improved overall mortality [10]. Recent high-quality review studies indicate that in-hospital mortality of SAP is associated with bleeding. However, the factors influencing the incidence of hemorrhagic complications in SAP are yet to be clarified [11].

Previous studies have analyzed the risk factors for pancreatic inflammation combined with abdominal bleeding and explored the risk factors for gastrointestinal bleeding in pancreatitis. These studies indicated that elevated CTSI and APACHE II scores are significantly associated with bleeding [12, 13]. However, most prior research consisted of case reports, small sample studies, and reviews, which failed to comprehensively assess the high-risk factors for bleeding complications in SAP. Although existing research mentions bleeding complications in SAP, the influencing factors remain unclear, and few studies compare gastrointestinal bleeding with abdominal bleeding. Therefore, we conducted a retrospective analysis of clinical data from SAP patients with bleeding complications admitted to our hospital. We aimed to identify the risk factors influencing SAP with bleeding complications, compare the differences between gastrointestinal and abdominal bleeding, and observe related prognoses. Our goal is to provide a reference for the early clinical identification of high-risk populations.

## Method

### Study design and participants

We retrospectively included clinical data of SAP patients hospitalized in the Department of Gastroenterology at the 1st affiliated Hospital, Jiangxi Medical College, Nanchang University, from January 2019 to December 2022. The data were obtained from the database maintained by the Department of Gastroenterology at the First Affiliated Hospital, which contains comprehensive information on patients diagnosed with SAP. This study was approved by the Ethics Committee of the 1st affiliated Hospital, Jiangxi Medical College, Nanchang University (No: 2011001). This study was designed and reported in accordance with the STROBE (Strengthening the Reporting of Observational Studies in Epidemiology) guidelines to ensure transparency and completeness in reporting observational research (Supplementary Material 1).

### Inclusion and exclusion criteria

#### Inclusion criteria

Complies with SAP diagnostics.

#### Exclusion criteria

(1). Patients with SAP due to post-ERCP procedures, trauma, pregnancy, and chronic pancreatitis (*n* = 23).

(2). Patients who experienced bleeding or underwent surgery and debridement before admission (*n* = 22).

(3). The patients had concurrent severe underlying conditions affecting the heart, lungs, liver, and kidneys (*n* = 20).

(4). Cases with missing data (*n* = 17).

(5). The patients had concurrent gastrointestinal and abdominal bleeding (*n* = 8).

#### Definitions

SAP was defined as AP with organ failure for more than 48 h; the definition of organ failure was according to the modified Marshall score system [2].

Sepsis is defined as a systemic inflammatory response syndrome caused by an explicit or suspected infection [14].

Infected pancreatic necrosis (IPN) was defined as enhanced CT suggestive of the “bubble sign” or positive culture of pancreatic necrotic tissue or drainage fluid for bacteria or fungus [2].

Abdominal compartment syndrome (ACS) is defined as when a sustained intra-abdominal pressure > 20 mmHg occurs with a new onset of organ dysfunction or failure [15].

Gastrointestinal bleeding is defined as active bleeding from hematemesis, melena, or nasogastric tube bleeding, all of which result in significant hemodynamic deterioration or a sharp drop in hemoglobin concentration by more than 2 g/dL, or a decrease in systolic blood pressure by 20mmHg accompanied by tachycardia due to bleeding [16].

Abdominal bleeding is defined as active bleeding characterized by blood drainage through a drain, new or worsening abdominal pain, distension without a drain, hemodynamic instability, or enhanced CT imaging showing high-density areas consistent with abdominal bleeding. This condition results in significant hemodynamic deterioration, a sharp drop in hemoglobin concentration by more than 2 g/dL, or a decrease in systolic blood pressure by 20mmHg accompanied by tachycardia due to bleeding [12].

The interval days to hemorrhage are defined as the number of days from the onset of the illness to the occurrence of bleeding.

The timing of debridement refers to the duration, measured in days, from the onset of SAP to the first endoscopic necrosectomy.

In this study, we focus on significant active bleeding that requires aggressive medical treatment, endoscopic hemostasis, angiographic intervention, or surgical hemostasis. Bleeding that occurs as oozing rather than active bleeding during endoscopic debridement, which can usually be controlled by electrocoagulation or ice saline irrigation, is not included. Minor bleeding, either observed or undetected, likely due to the erosion of small vessels by activated proteolytic enzymes, generally does not require special treatment. Postoperative bleeding following surgery is rare in this retrospective study, with only two cases reported. These minor bleedings, bleeding during endoscopic debridement, and postoperative bleeding are beyond the scope of this discussion.

### Observational indicators

For the purpose of a more precise analysis of factors influencing hemorrhage in SAP, all pertinent data pertaining to laboratory tests, imaging, scoring systems, complications, and interventions recorded within the 3 days preceding the hemorrhagic event were collected. The collected parameters included white blood cell count (WBC), platelet count (PLT), albumin level (ALB), blood urea nitrogen level (BUN), creatinine level (Cr), prothrombin time (PT), activated partial thromboplastin time (APTT), C-reactive protein (CRP), procalcitonin (PCT), lactate level, CTSI score, APACHE II score, infected pancreatic necrosis (IPN), organ failure status, sepsis, blood culture results, fungal infection, abdominal compartment syndrome (ACS), interventions such as percutaneous catheter drainage (PCD), endoscopic necrosectomy (EN), transluminal endoscopic necrosectomy (TEN), percutaneous endoscopic necrosectomy (PEN), endoscopic retrograde cholangiopancreatography (ERCP), mechanical ventilation, and continuous renal replacement therapy (CRRT). Therefore, these data can be considered pre-bleeding-related data, providing a more accurate reflection of the patient’s condition before the onset of bleeding. Clinical data recorded included gender, age, body mass index (BMI), smoking and drinking habits, history of diabetes and hypertension, etiology, type of bleeding, bleeding rate, rebleeding rate, mortality rate, hospitalization duration, and the need for surgery. However, details regarding hemostatic treatment measures are not within the scope of this study.

### Hemostatic intervention

For SAP patients with bleeding, our center typically employs a multidisciplinary team (MDT) approach. For patients with minor hematemesis, melena, or minor bloody drainage, we initially opted for conservative treatment with hemostatic medications. If significant hematochezia or hematemesis is observed, suggesting potential gastrointestinal bleeding, we prefer endoscopic hemostasis combined with pharmacotherapy. Should the bleeding persist, angiography and subsequent treatment are considered. If an angiographic intervention fails to control the bleeding, surgical hemostasis is then contemplated. For patients with substantial bloody drainage from the abdominal cavity, indicating potential abdominal bleeding, angiography, and treatment are generally prioritized. If bleeding remains uncontrolled, surgical hemostasis or conservative medical treatment is then pursued.

### Statistical analysis

In the study, we performed 1:4 nearest neighbor matching without replacement using a caliper value of 0.1 in the propensity score matching (PSM) Matching plug-in in SPSS 26.0 software for PSM. For normally distributed quantitative data, we described it by mean ± standard deviation; for non-normally distributed quantitative data, we used the median and interquartile range (IQR). The t-test and Mann–Whitney U test was used for quantitative data comparison. Qualitative data were described by frequency and percentage and analyzed using the Chi-square test and Fisher’s exact probability method. Variables with significant differences between the two groups were included in univariate and multivariate logistic regression analysis (backward method) to identify independent influencing factors of SAP-associated hemorrhage. Results were expressed as odds ratio (OR) with a 95% confidence interval (CI). Based on the factors identified from the multivariate logistic regression analysis, we constructed a nomogram prediction model for predicting SAP bleeding using the rms package in R4.3.2 software. We also drew the ROC curve and calculated the area under the curve (AUC). Statistical analysis was completed using SPSS 26.0 software, and *P* < 0.05 was considered statistically significant.

## Results

### Flow chart

**Fig. 1:**
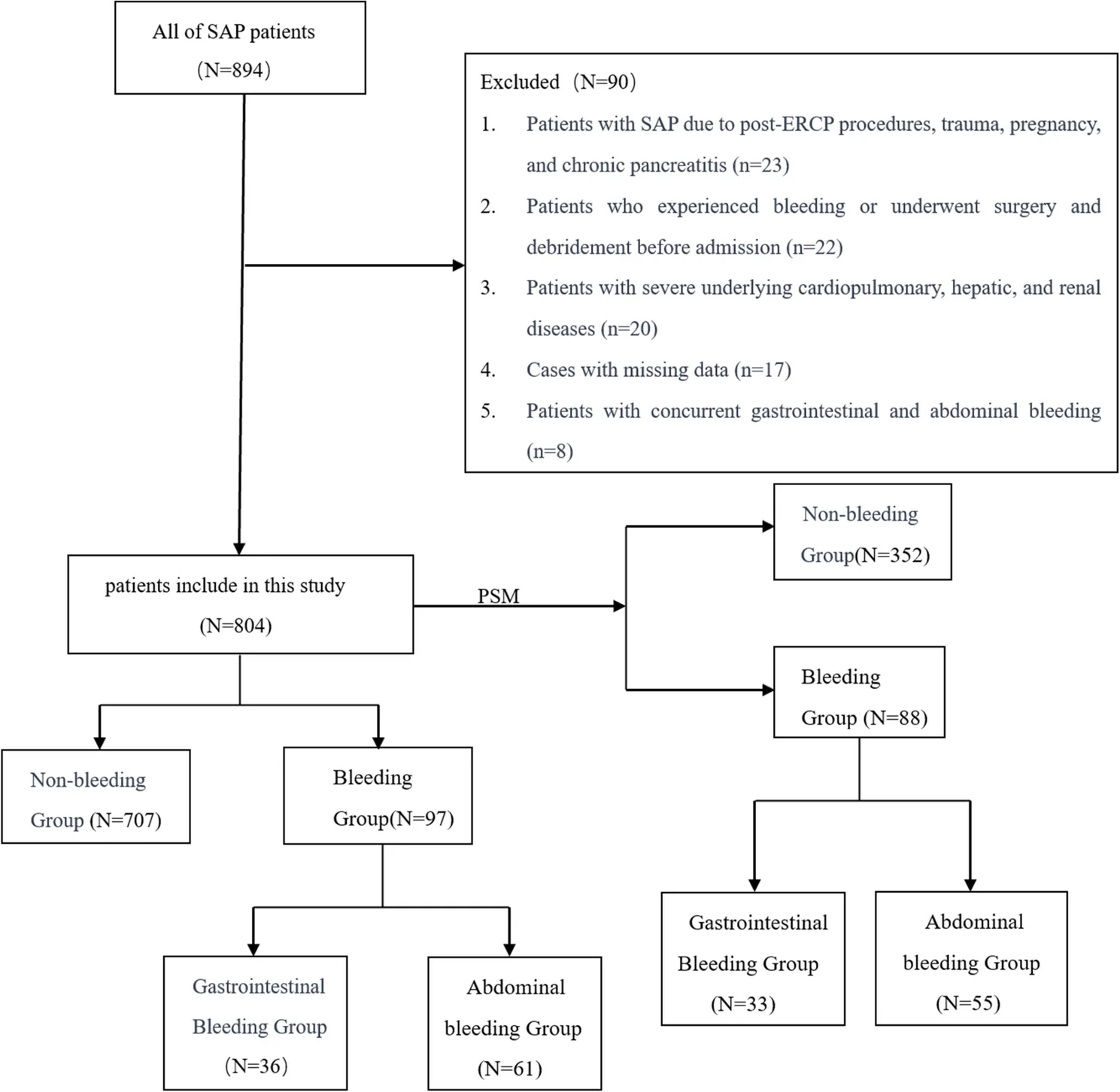
Flowchart of the procedure ; SAP: Severe Acute Pancreatitis SAP: Severe Acute Pancreatitis

This study retrospectively analyzed the clinical data of 894 SAP patients hospitalized in our department from January 2019 to December 2022. After applying the inclusion criteria, 90 cases were excluded, leaving 804 SAP patients in the study. Among these patients, 97 (12.1%) experienced bleeding complications, with 36 cases (37.1%) of gastrointestinal bleeding and 61 cases (62.9%) of abdominal cavity bleeding. The study flowchart is shown in Fig. 1.

### Propensity score matching method

In the study, we applied the propensity score matching method to effectively balance the baseline differences between the bleeding and non-bleeding groups, thereby reducing the influence of confounding variables and enhancing the reliability of the results. Using a caliper value of 0.1, we performed 1:4 nearest neighbor matching without replacement. This resulted in 440 successfully matched SAP patients, including 88 patients in the bleeding group and 352 patients in the non-bleeding group. Before matching, we compared the two groups in terms of gender, age, BMI, smoking and drinking habits, diabetes, hypertension, and etiology. There was a significant statistical difference in age between the two groups before matching (*P* = 0.038). After matching, the comparison indicated that all included variables did not reach statistical significance (*P* > 0.05) between the two groups, confirming the comparability of the data. Specific details are shown in Table 1.

**Table 1:** Baseline characteristics of bleeding and non-bleeding groups in SAP patients before and after PSM analysis

Characteristics	Before Matching		Pvalue	After Matching		Pvalue
	Bleeding Groups(*n* = 97)	NonBleeding Groups(*n* = 707)		Bleeding Groups(*n* = 88)	NonBleeding Groups(*n* = 352)	
Age, years, (SD)	48 ± 13.8	52 ± 16.8	0.038	49 ± 13.3	49 ± 15	0.946
Sex, n (%)			0.751			0.866
Male	55 (56.7%)	417 (59.0%)		51(58.0%)	198 (56.2%)	
Female	42 (43.3%)	290 (41.0%)		37 (42.0%)	154 (43.8%)	
BMI, kg/m^2^, (SD)	25 ± 3.77	25 ± 4.14	0.605	25 ± 3.72	25 ± 4.62	0.719
Smoking, n(%)	33 (34.0%)	230 (32.5%)	0.859	29 (33.0%)	119 (33.8%)	0.980
Drinking, n(%)	34 (35.1%)	249 (35.2%)	1.000	31 (35.2%)	118(33.5%)	0.860
Diabetes, n(%)	14 (14.4%)	104 (14.7%)	1.000	14 (15.9%)	55 (15.6%)	1.000
Hypertension, n(%)	27 (27.8%)	191 (27.0%)	0.961	24 (27.3%)	97 (27.6%)	1.000
Etiology, n (%)						
Biliary	47 (48.5%)	338 (47.8%)	0.991	40 (45.5%)	159 (45.2%)	1.000
Hyperlipidemia	41 (42.3%)	296 (41.9%)	1.000	40 45.5%)	159 (45.2%)	1.000
Alcoholic	11 (11.3%)	86 (12.2%)	0.946	11(12.5%)	45 (12.8%)	1.000
Others	6 ( 6.2%)	36 ( 5.1%)	0.833	5 ( 5.7%)	17 ( 4.8%)	0.956

*mean* (SD), *SD* Standard deviation, *median* (IQR), or n (%), *IQR* Interquartile range, *BMI* body mass index, *SAP* Severe Acute Pancreatitis

### Analysis of baseline characteristics between the bleeding and non-bleeding groups

The baseline characteristics of the two patient groups are presented in Table 2, encompassing demographics, medical history, laboratory indicators, complications, imaging data, and clinical scoring systems. The results reveal that the median age of all patients was 48 years, with males accounting for 56.6%. Etiologically, biliary origin and hypertriglyceridemia were the most common causes of SAP, each constituting 45.2%. The bleeding group exhibited significantly lower levels of WBC and ALB compared to the non-bleeding group, while BUN, Cr, PT, APTT, PCT, and lactate levels were markedly higher. Additionally, the bleeding group had a significantly higher incidence of renal failure, multiple organ failure, and pancreatic necrosis involving ≥ 50% of the pancreas. Furthermore, the number of patients with CTSI scores and APACHE II scores ≥ 15 was significantly greater in the bleeding group. Cases of IPN, sepsis, positive blood cultures, fungal infections, and ACS were also significantly more prevalent in the bleeding group. These baseline variables exhibited statistically significant differences between the two groups (*P* < 0.05). However, there were no significant differences between the bleeding and non-bleeding groups regarding gender, age, BMI, smoking and alcohol consumption, diabetes, hypertension, etiology, PLT, CRP, and respiratory failure (*P* > 0.05).

**Table 2:** Demographic and clinical characteristics of bleeding and non-bleeding groups

	All Patients (*n* = 440)	Bleeding Groups(*n* = 88)	Non-Bleeding Groups(*n* = 352)	Pvalue
Age, years, IQR	48(37,59)	49(38,56)	47(37,60)	0.967
Sex, n (%)				0.773
Male	249 (56.6%)	51 (58.0%)	198 (56.2%)	
Female	191 (43.4%)	37 (42.0%)	154 (43.8%)	
BMI, kg/m^2^, IQR	24.3(22.9,26.8)	24.5(22.9,26.8)	24.3(22.9,26.8)	0.867
Smoking, n(%)	148 (33.6%)	29 (33.0%)	119 (33.8%)	0.880
Drinking, n(%)	149 (33.9%)	31 (35.2%)	118 (33.5%)	0.762
Diabetes, n(%)	69 (15.7%)	14 (15.9%)	55 (15.6%)	0.948
Hypertension, n(%)	121 (27.5%)	24 (27.3%)	97 (27.6%)	0.957
Etiology, n (%)				
Biliary	199 (45.2%)	40 (45.5%)	159 (45.2%)	0.962
Hyperlipidemia	199 (45.2%)	40 (45.5%)	159 (45.2%)	0.962
Alcoholic	56 (12.7%)	11 (12.5%)	45 (12.8%)	0.943
Others	22 ( 5.0%)	5 ( 5.7%)	17 ( 4.8%)	0.743
Laboratory data				
WBC(10^9^/L), IQR	13.0(9.4,18.4)	11.1(7.5, 18.8)	13.7(10.2, 18.3)	0.010
PLT (10^9^/L), IQR	198(148,259)	223(139, 302)	190(149, 251)	0.109
ALB( g/L), IQR	33.6(29.7,37.6)	32.2(27.5, 36.1)	33.8(30.3, 38.2)	0.001
BUN ( mmol/L), IQR	7.0(4.7,11.5)	11.4(6.4, 20.1)	6.6(4.4, 10.1)	< 0.001
Cr (umol/L), IQR	85.2(59.9,168.0)	143.3(72.1,273.9)	80.1(57.2, 146.2)	< 0.001
PT (s), IQR	12.6(11.7,13.9)	13.6(12.4, 14.9)	12.5(11.6, 13.6)	< 0.001
APTT (s), IQR	28.2(25.7,32.1)	29.5(26.1, 36.0)	28.1(25.6, 31.6)	0.049
CRP (mg/L), IQR	124.0(72.7,183.0)	120.5(59.6,200.7)	124.0(75.8,181.0)	0.989
PCT (ng/ml), IQR	0.8(0.3,2.3)	1.4(0.5, 5.5)	0.7(0.3, 2.1)	< 0.001
Lactate( mmol/L), IQR	1.7(1.2,2.4)	2.1(1.5, 2.9)	1.7(1.2, 2.3)	< 0.001
Organ failures, *n* (%)				
Respiratory failures	405(92.0%)	83(94.3%)	322 (91.5%)	0.378
Renal failures	165 (37.5%)	57 (64.8%)	108 (30.7%)	< 0.001
MOF	164 (37.3%)	65 (73.9%)	99 (28.1%)	< 0.001
CTSI score, IQR	6.0(5.0,8.0)	10.0(8.0, 10.0)	6.0(4.0, 8.0)	< 0.001
APACHEIIscore ≥ 15,n(%)	123 (28.0%)	71 (80.7%)	52 (14.8%)	< 0.001
Pancreatic necrosis area, n(%)				< 0.001
≤ 30%	157 (35.7%)	10 (11.4%)	147 (41.8%)	
30–50%	101 (23.0%)	22 (25.0%)	79 (22.4%)	
≥ 50%	87 (19.8%)	52 (59.1%)	35 ( 9.9%)	
IPN, n(%)	135 (30.7%)	71 (80.7%)	64 (18.2%)	< 0.001
Sepsis, n(%)	114 (25.9%)	52 (59.1%)	62 (17.6%)	< 0.001
Positive blood culture, n(%)	56 (12.7%)	32 (36.4%)	24 ( 6.8%)	< 0.001
Fungal infection, n(%)	47 (10.7%)	24 (27.3%)	23 ( 6.5%)	< 0.001
ACS, n(%)	56 (12.7%)	30 (34.1%)	26 ( 7.4%)	< 0.001

*median* (IQR), or n (%), *IQR* Interquartile range, *WBC* white blood count, *PLT* platelet, *PT* prothrombin time, *APTT* activated partial thromboplastin time, *ALB* serum albumin, *Cr* serum creatinine, *BUN* blood urea nitrogen, *PCT* procalcitonin, *CRP* C-reactive protein, *MOF* Multiple organ failure(*n* ≥ 2), *CTSI* computed tomography severity index, *APACHE II* Acute Physiology and Chronic Health Evaluation II, *IPN* Infected Pancreatic Necrosis, *ACS* abdominal compartment syndrome, *ICU* intensive care unit

### Pre-bleeding interventions and prognostic information in the bleeding and non-bleeding groups

To further investigate the relationship between pre-bleeding interventions and bleeding, as well as their impact on prognosis, we collected clinical data on the timing and frequency of endoscopic debridement before hemorrhage, different access routes, the necessity for ERCP, usage rates of PCD, mechanical ventilation, and CRRT, hospitalization duration, and mortality rates. According to the data in Table 3, most initial endoscopic debridement occurred after 30 days, with no significant difference between the two groups (*P* = 0.233). Regarding the frequency of endoscopic debridement, although the bleeding group had a higher number of debridement procedures compared to the non-bleeding group (20.4% vs. 15%), there was no significant difference between the two groups in terms of single versus multiple debridement procedures (*P* = 0.477). Regarding EN, although the bleeding group had more EN procedures than the non-bleeding group (21.6% vs. 15.1%), the difference was not statistically significant (*P* = 0.138). Similarly, there was no significant difference between the two groups concerning different access routes for EN, including TEN and PEN (*P* > 0.05). However, the usage rates of PCD, mechanical ventilation, and CRRT were significantly higher in the hemorrhage group compared to the non-bleeding group (*P* < 0.001), indicating a significant statistical difference between the two groups. Additionally, the impact on prognosis was evaluated using indicators such as length of hospital stay, in-hospital mortality, and the need for surgical intervention. The median ICU stay and the total median hospital stay in the bleeding group were significantly longer than those in the non-bleeding group (30 vs. 7, 48 vs. 18, *P* < 0.001), indicating a longer hospital stay for patients in the bleeding group. The number of SAP patients requiring surgical intervention was significantly higher in the bleeding group compared to the non-bleeding group (21.6% vs. 0.9%, *P* < 0.001). Furthermore, in-hospital mortality was significantly higher in the bleeding group than in the non-bleeding group (46.6% vs. 6.5%, *P* < 0.001), indicating that bleeding significantly increases the in-hospital mortality rate of SAP patients.

**Table 3:** Pre-bleeding interventions and prognostic information in the bleeding and no-bleeding groups

	All Patients (*n* = 440)	Bleeding Groups(*n* = 88)	Non-Bleeding Groups(*n* = 352)	*P* value
Timing of debridement, *n*(%)				0.233
≤ 30 days	6 ( 1.4%)	1 ( 1.1%)	5 ( 1.4%)	
30–60 days	49 (11.1%)	15 (17.0%)	34 ( 9.7%)	
≥ 60 days	16 ( 3.6%)	2 ( 2.3%)	14 ( 4.0%)	
EN, n(%)	72 (16.4%)	19 (21.6%)	53 (15.1%)	0.138
TEN, n(%)	31 ( 7.0%)	9 (10.2%)	22 ( 6.2%)	0.192
PEN, n(%)	45 (10.2%)	11 (12.5%)	34 ( 9.7%)	0.431
Frequency of Debridement, n(%)				0.477
1	20 ( 4.5%)	5 ( 5.7%)	15 ( 4.3%)	
2	20 ( 4.5%)	4 ( 4.5%)	16 ( 4.5%)	
≥ 3	31 ( 7.0%)	9 (10.2%)	22 ( 6.2%)	
PCD, n(%)	137 (31.1%)	46 (52.3%)	91 (25.9%)	< 0.001
ERCP, n(%)	37 ( 8.4%)	5 ( 5.7%)	32 ( 9.1%)	0.303
CRRT, n(%)	99 (22.5%)	42 (47.7%)	57 (16.2%)	< 0.001
Mechanical ventilation, n(%)	172 (39.1%)	68 (77.3%)	104 (29.5%)	< 0.001
Need forsurgery, n(%)	22 ( 5.0%)	19 (21.6%)	3 ( 0.9%)	< 0.001
Hospital Stay, days, IQR	21(12,40)	48(26, 73)	18(12, 31)	< 0.001
Hospital stay in ICU, days, IQR	9(3,21)	30(14, 49)	7(1, 13)	< 0.001
Mortality, n(%)	64 (14.5%)	41 (46.6%)	23 ( 6.5%)	< 0.001

*PCD *percutaneous catheter drainage, *EN* Endoscopic Necrosectomy, *TEN* Transluminal Endoscopic Necrosectomy, *PEN* Percutaneous Endoscopic Necrosectomy, *ERCP* Endoscopic Retrograde Cholangiopancreatography, *CRRT* Continuous Renal Replacement Therapy

### Univariate and multivariate logistic regression analyses

In univariate and multivariate logistic regression analysis (Table 4), we identified significant factors associated with SAP bleeding. Variables that were significantly different between the bleeding and non-bleeding groups in Tables 2 and 3 were included in the univariate and multivariate logistic regression analysis. The results indicated that CTSI score (OR: 1.771, 95% CI: 1.434–2.187, *P* < 0.001), IPN(OR: 4.407, 95% CI: 1.854–10.476, *P* < 0.001), APACHE II score ≥ 15 (OR: 12.323, 95% CI: 5.015–30.282, *P* < 0.001), ACS(OR: 3.685, 95% CI: 1.462–9.287, *P* = 0.006), and CRRT(OR: 0.251, 95% CI: 0.094–0.670, *P* = 0.006) were independent influencing factors of SAP-related bleeding.

**Table 4:** Univariate and multivariate logistic regression analysis of factors affecting bleeding in SAP patients

	Univariate Analysis		Multivariate Analysis	
	OR (95% CI)	*P* value	OR (95% CI)	*P* value
WBC(10^9^/L)	0.974(0.938,1.010)	0.158	-	-
ALB( g/L)	0.992(0.961,1.025)	0.638	-	-
PT (s)	1.144(1.056,1.240)	< 0.001	1.064(0.965,1.173)	0.214
APTT (s)	1.017(0.999,1.035)	0.060	0.974(0.937,1.012)	0.179
PCT (ng/ml)	1.012(0.998,1.025)	0.097	-	-
BUN ( mmol/L)	1.091(1.059,1.123)	< 0.001	1.046(0.994,1.101)	0.081
Cr (umol/L)	1.003(1.001,1.004)	0.001	-	-
Lactate( mmol/L)	1.323(1.153,1.518)	< 0.001	1.160(0.943,1.426)	0.161
Renal failures	4.154(2.538,6.798)	< 0.001	-	-
MOF	7.222( 4.254,12.261)	< 0.001	-	-
CTSI score	1.996( 1.706, 2.336)	< 0.001	1.771(1.434,2.187)	< 0.001
APACHE II score ≥ 15	24.095(13.150,44.151)	< 0.001	12.323(5.015,30.282)	< 0.001
Pancreatic necrosis area				
≤ 30%	0.179( 0.090, 0.357)	< 0.001	-	-
30–50%	1.152( 0.669, 1.984)	0.610	-	-
≥ 50%	13.083( 7.548,22.674)	< 0.001	-	-
IPN	18.794(10.371,34.057)	< 0.001	4.407(1.854,10.476)	< 0.001
Sepsis	6.756( 4.074,11.204)	< 0.001	0.496(0.198, 1.242)	0.134
Positive blood culture	7.810( 4.284,14.235)	< 0.001	-	-
Fungal infection	5.364( 2.853,10.087)	< 0.001	-	-
ACS	6.485( 3.578,11.756)	< 0.001	3.685(1.462, 9.287)	0.006
PCD	3.141( 1.941, 5.085)	< 0.001	-	-
CRRT	4.725( 2.851, 7.833)	< 0.001	0.251(0.094, 0.670)	0.006
Mechanical ventilation	8.108( 4.684,14.034)	< 0.001	-	-

*SAP* Severe Acute Pancreatitis, *WBC* white blood count, *PT* prothrombin time, *APTT* activated partial thromboplastin time, *ALB* serum albumin, *Cr* serum creatinine, *BUN* blood urea nitrogen, *PCT* procalcitonin, *MOF* Multiple organ failure(*n* ≥ 2), *CTSI* computed tomography severity index, *APACHE II* Acute Physiology and Chronic Health Evaluation II, *IPN* Infected Pancreatic Necrosis, *ACS* abdominal compartment syndrome, *PCD* percutaneous catheter drainage, *CRRT* Continuous Renal Replacement Therapy

### Construction of a nomogram prediction mode

**Fig. 2:**
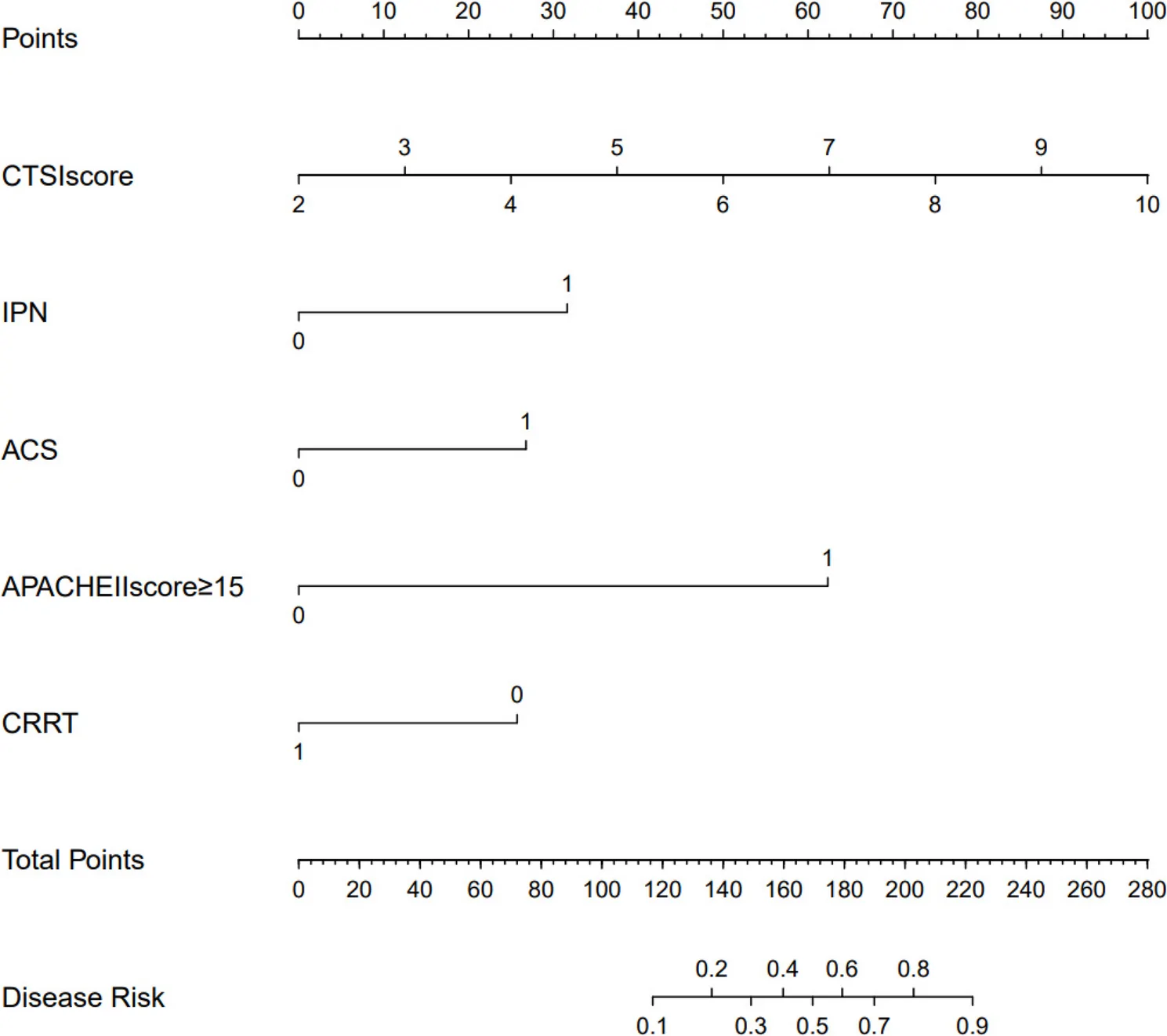
Nomogram prediction model for hemorrhage in SAP patients; SAP: severe acute pancreatitis

To predict the risk of SAP bleeding, a nomogram prediction model was developed based on the five statistically significant factors identified in the multivariate logistic regression analysis using R. As shown in Fig. 2, the nomogram prediction model revealed that CTSI score, IPN, APACHE II score ≥ 15, and ACS were independent risk factors for bleeding, with odds ratios (OR) greater than 1 for these variables (CTSI score OR: 1.771, IPN OR: 4.407, APACHE II score ≥ 15 OR: 12.323, ACS OR: 3.685). The longer line segments for CTSI scores and APACHE II scores ≥ 15 in the nomogram indicate their high impact on the predictive model. This suggests that the risk of hemorrhage in SAP is closely associated with the severity of the condition, as reflected by these scores. Conversely, CRRT is negatively correlated with the risk of hemorrhage (OR: 0.251, *P* = 0.006), suggesting it may serve as a protective factor for SAP patients, helping to reduce the likelihood of hemorrhage. The nomogram illustrates that when the total score of the five variables reaches 220 points, the probability of predicting hemorrhage risk in SAP patients is 90%.

### ROC curve

**Fig. 3:**
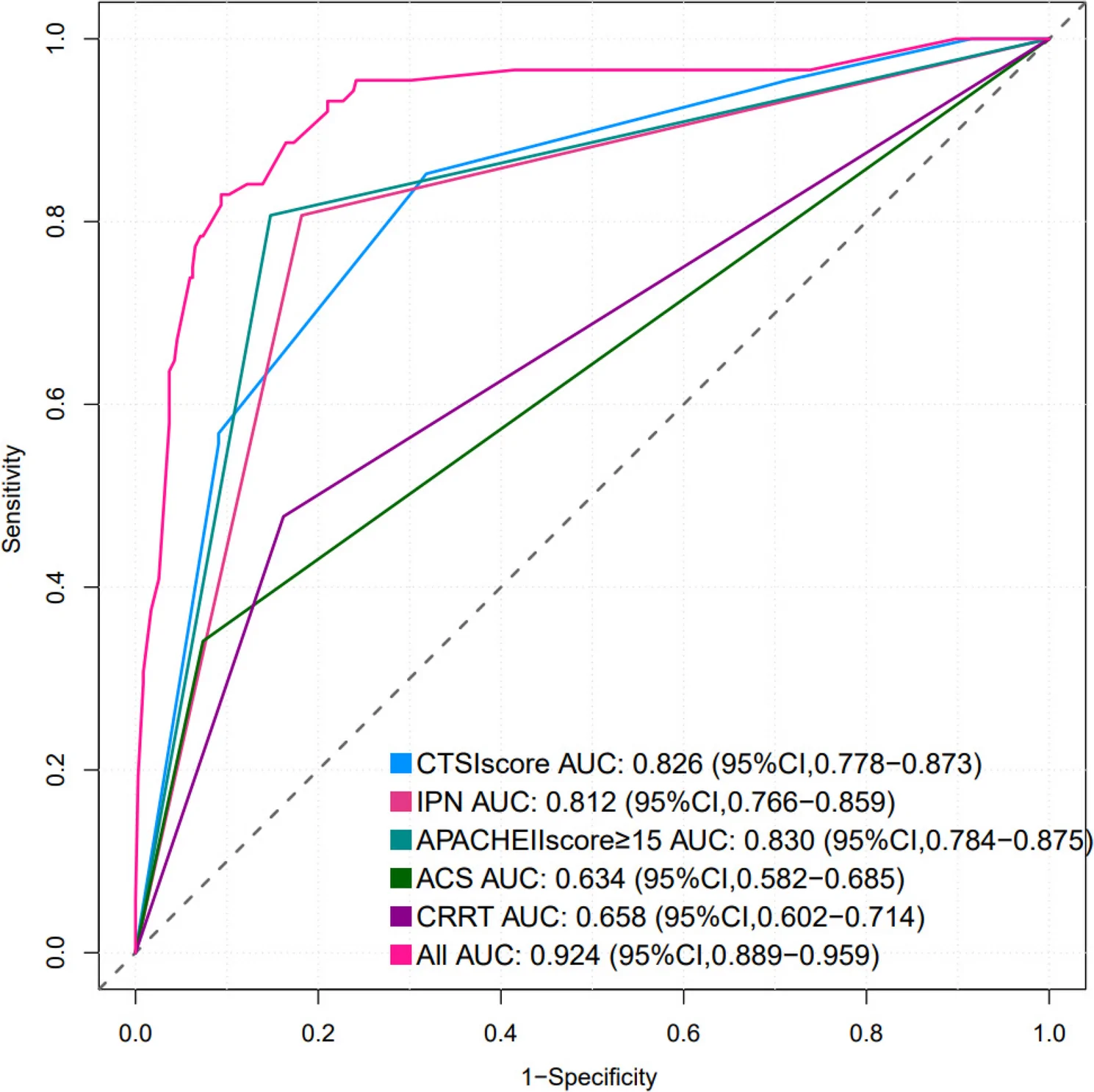
AUCs of CTSI score model, IPN model, APACHE II score ≥ 15 model, ACS model, CRRT model, and combined model for the diagnosis of bleeding in the study sample. The combined model displayed the best diagnostic performance for the prediction of bleeding

By including the five independent influencing factors, we drew the ROC curve as shown in Fig. 3. When considering each factor alone, the area under the curve (AUC) values were as follows: CTSI score, 0.826 (95% CI: 0.778–0.873); IPN, 0.812 (95% CI: 0.776–0.859); APACHE II score ≥ 15, 0.830 (95% CI: 0.784–0.875); ACS, 0.634 (95% CI: 0.582–0.685); and CRRT, 0.658 (95% CI: 0.602–0.714). When the five influencing factors were combined into a single prediction model, the AUC for the combined prediction of bleeding risk was 0.924 (95% CI: 0.889–0.959), indicating that the combined prediction model had the best diagnostic performance.

### Comparative analysis of gastrointestinal bleeding and abdominal bleeding

To further investigate the differences between gastrointestinal bleeding and abdominal bleeding, we conducted a detailed subgroup analysis (see Tables 5 and 6). Among the 88 SAP patients with hemorrhagic complications, there were 33 cases of gastrointestinal bleeding and 55 cases of abdominal bleeding. The median time from onset of SAP to bleeding was 30 days. Baseline characteristics, including gender, age, BMI, medical history, etiology, laboratory indicators, organ failure, scoring systems, pancreatic necrosis, complications, and the interval time to bleeding, showed no significant differences between the gastrointestinal bleeding group and the abdominal bleeding group (*P* > 0.05). Further comparisons of pre-bleeding interventions and prognosis details, including debridement timing, endoscopic debridement, frequency of debridement, ERCP, mechanical ventilation, CRRT, rebleeding rates, hospitalization duration, rebleeding interval, and mortality rates, also revealed no significant differences between the two groups. However, notable differences were observed in PCD, the need for surgery, and the rebleeding interval. The abdominal bleeding group had a significantly higher number of patients receiving PCD compared to the gastrointestinal bleeding group (63.6% vs. 33.3%, *P* = 0.006). Additionally, the abdominal bleeding group had a significantly higher number of patients requiring surgery (29.1% vs. 9.1%, *P* = 0.027), and the rebleeding interval was significantly shorter in the abdominal bleeding group compared to the gastrointestinal bleeding group (4 vs. 24, *P* = 0.031).

**Table 5:** Baseline analysis of gastrointestinal and abdominal bleeding subgroups

	Bleedin Groups (*n* = 88)	Gastrointestinal Bleeding (*n* = 33)	Abdominal Bleeding(*n* = 55)	Pvalue
Age, years, IQR	49(38,56)	49(41, 56)	49(37, 56)	0.669
Sex, n (%)				0.696
Male	51 (58.0%)	20 (60.6%)	31 (56.4%)	
Female	37 (42.0%)	13 (39.4%)	24 (43.6%)	
BMI, kg/m^2^, IQR	24.5(22.9,26.8)	25.4(22.9, 28.7)	24.2(22.8, 25.6)	0.139
Smoking, n(%)	29 (33.0%)	10 (30.3%)	19 (34.5%)	0.682
Drinking, n(%)	31 (35.2%)	11 (33.3%)	20 (36.4%)	0.773
Diabetes, n(%)	14 (15.9%)	6 (18.2%)	8 (14.5%)	0.652
Hypertension, n(%)	24 (27.3%)	9 (27.3%)	15 (27.3%)	1.000
Etiology, n (%)				
Biliary	40 (45.5%)	14 (42.4%)	26 (47.3%)	0.658
Hyperlipidemia	40 (45.5%)	18 (54.5%)	22 (40.0%)	0.185
Alcoholic	11 (12.5%)	4 (12.1%)	7 (12.7%)	0.934
Others	5 ( 5.7%)	1 ( 3.0%)	4 ( 7.3%)	0.405
Laboratory data				
WBC(10^9^/L), IQR	11.1(7.5,18.8)	10.2(7.6, 13.6)	12.5(7.3, 19.0)	0.617
PLT (10^9^/L), IQR	223(139,302)	218(142, 276)	241(138, 336)	0.343
ALB( g/L), IQR	32.2(27.5,36.1)	32.9(28.6, 36.3)	30.7(27.1, 35.9)	0.326
PT (s), IQR	13.7(12.4,14.9)	13.0(12.0, 14.7)	13.9(12.5, 15.2)	0.152
APTT (s), IQR	29.5(26.1,36.0)	28.4(24.2, 35.3)	29.9(26.5, 37.0)	0.151
CRP (mg/L), IQR	120.5(59.7,200.7)	88.2(37.8, 178.7)	129.8(86.0,203.8)	0.151
PCT (ng/ml), IQR	1.4(0.5,5.5)	1.0(0.4, 4.9)	1.4(0.7, 5.5)	0.309
BUN ( mmol/L), IQR	11.5(6.5,20.1)	11.2(5.9, 22.6)	11.7(7.2, 19.0)	0.853
Cr (umol/L), IQR	143.3(72.1,273.9)	151.8(75.0,220.3)	139.7(67.6,295.9)	0.766
Lactate( mmol/L), IQR	2.1(1.5,2.9)	2.1(1.5, 2.5)	2.2(1.5, 3.2)	0.386
Organ failures, *n* (%)				
Respiratory failures	83 (94.3%)	31 (93.9%)	52 (94.5%)	0.905
Renal failures	57 (64.8%)	21 (63.6%)	36 (65.5%)	0.863
MOF	65 (73.9%)	23 (69.7%)	42 (76.4%)	0.491
CTSI score, IQR	10(8, 10)	10(8, 10)	10(8, 10)	0.257
APACHEIIscore ≥ 15,n(%)	71 (80.7%)	24 (72.7%)	47 (85.5%)	0.143
Pancreatic necrosis area, n(%)				0.491
≤ 30%	10 (11.4%)	5 (15.2%)	5 ( 9.1%)	
30–50%	22 (25.0%)	6 (18.2%)	16 (29.1%)	
≥ 50%	52 (59.1%)	19 (57.6%)	33 (60.0%)	
IPN, n(%)	71 (80.7%)	27 (81.8%)	44 (80.0%)	0.834
Sepsis, n(%)	52 (59.1%)	21 (63.6%)	31 (56.4%)	0.502
Positive blood culture, n(%)	32 (36.4%)	15 (45.5%)	17 (30.9%)	0.170
Fungal infection, n(%)	24 (27.3%)	12 (36.4%)	12 (21.8%)	0.138
ACS, n(%)	30 (34.1%)	15 (45.5%)	15 (27.3%)	0.082
Bleeding intervals, days, IQR	30(18,45)	29(18, 42)	30(21, 54)	0.507

*median* (IQR), or n (%), *IQR* Interquartile range, *WBC* white blood count, *PLT* platelet, *PT* prothrombin time, *APTT* activated partial thromboplastin time, *ALB* serum albumin, *Cr* serum creatinine, *BUN* blood urea nitrogen, *PCT* procalcitonin, *CRP* C-reactive protein, *MOF* Multiple organ failure (*n* ≥ 2), *CTSI* computed tomography severity index, *APACHE II* Acute Physiology and Chronic Health Evaluation II, *IPN* Infected Pancreatic Necrosis, *ACS* abdominal compartment syndrome

**Table 6:** Gastrointestinal and abdominal bleeding in pre-bleeding interventions and prognostic details

	Bleedin Patients (*n* = 88)	Gastrointestinal Bleeding (*n* = 33)	Abdominal Bleeding(*n* = 55)	Pvalue
Timing of debridement, *n*(%)				0.143
≤ 30 days	1 ( 1.1%)	0 ( 0.0%)	1 ( 1.8%)	
30–60 days	15 (17.0%)	6 (18.2%)	9 (16.4%)	
≥ 60 days	2 ( 2.3%)	0 ( 0.0%)	2 ( 3.6%)	
EN, n(%)	19 (21.6%)	6 (18.2%)	13 (23.6%)	0.547
TEN, n(%)	9 (10.2%)	3 ( 9.1%)	6 (10.9%)	0.785
PEN, n(%)	11 (12.5%)	3 ( 9.1%)	8 (14.5%)	0.454
Frequency of Debridement, n(%)				0.454
1	5 ( 5.7%)	2 ( 6.1%)	3 ( 5.5%)	
2	4 ( 4.5%)	1 ( 3.0%)	3 ( 5.5%)	
≥ 3	9 (10.2%)	3 ( 9.1%)	6 (10.9%)	
PCD, n(%)	46 (52.3%)	11 (33.3%)	35 (63.6%)	0.006
ERCP, n(%)	5 ( 5.7%)	2 ( 6.1%)	3 ( 5.5%)	0.905
CRRT, n(%)	42 (47.7%)	15 (45.5%)	27 (49.1%)	0.741
Mechanical ventilation, n(%)	68 (77.3%)	25 (75.8%)	43 (78.2%)	0.793
Need forsurgery, n(%)	19 (21.6%	3 ( 9.1%)	16 (29.1%)	0.027
Rebleeding, n(%)	23 (26.1%)	9 (27.3%)	14 (25.5%)	0.851
Rebleeding intervals, days, IQR	7(3,24)	24(7, 25)	4(0, 10)	0.031
Hospital Stay, days, IQR	48(26,73)	61(29, 83)	42(25, 65)	0.226
Hospital stay in ICU, days, IQR	30(14,49)	32(13, 53)	29(14, 46)	0.518
Mortality, n(%)	41 (46.6%)	12 (36.4%)	29 (52.7%)	0.136

*median *(IQR), or n (%), *IQR* Interquartile range, *PCD* percutaneous catheter drainage, *EN* Endoscopic Necrosectomy, *TEN* Transluminal Endoscopic Necrosectomy, *PEN* Percutaneous Endoscopic Necrosectomy, *ERCP *Endoscopic Retrograde Cholangiopancreatography, *CRRT* Continuous Renal Replacement Therapy, *ICU* intensive care unit

## Discussion

The incidence of hemorrhagic complications in SAP has been reported to range from 1.2% to 14.5% in the literature [6]. A domestic study found that the bleeding rate could be as high as 15% in patients with acute necrotizing pancreatitis [17], while a foreign study reported that 6.2% (28/449) of patients experienced hemorrhages, with an average bleeding time of 27 days into the disease course [4]. Due to the diverse clinical manifestations of AP-related bleeding, its incidence varies significantly. For example, a bleeding rate of 11.5% was reported in a study of 539 AP cases [5], and another study on SAP indicated that 24 (13%) cases had bleeding complications, with an average duration of 27 days before bleeding occurred [16]. In our study, out of 804 SAP cases, 97 (12.1%) developed hemorrhages, with a median bleeding time of 30 days. Most of these cases occurred in the later stages of the pancreatitis course, consistent with previous studies.

The in-hospital mortality rate of hemorrhage in this study was 46.6%. Previous studies have shown that hemorrhages are associated with significant mortality rates, regardless of the type of bleeding, with estimated in-hospital mortality rates ranging from 34% to 52% [18, 19]. However, a multicenter prospective cohort study [7] found that, after controlling for related confounding factors, hemorrhagic events did not significantly increase mortality rates. Similarly, other studies concluded that hemorrhagic complications themselves had little relationship with mortality rates [4]. Conversely, recent studies have reported that SAP combined with hemorrhage is associated with poor prognosis and that bleeding increases in-hospital mortality rates [16]. In this study, we analyzed the effect of hemorrhage on in-hospital mortality rates and found that the mortality rate of patients with hemorrhage was significantly higher than that of non-bleeding patients (46.6% vs. 6.5%, *P* < 0.001). We believe that hemorrhagic events significantly increase the in-hospital mortality rate of SAP patients. The CTSI score and APACHE II score are useful for assessing the outcome of AP at an early stage and predicting prognosis [11, 20]. In our results, the CTSI score, APACHE II score, and complication incidence rate of the bleeding group were significantly higher than those of the non-bleeding group. This suggests that patients in the bleeding group had more severe conditions and a worse prognosis, which is similar to the findings of a domestic study [21]. Hemorrhagic events represent a secondary hit for patients, particularly those with SAP and organ dysfunction. Hemorrhage exacerbates the condition, prolongs hospitalization, and increases mortality rates. Notably, 41 (46.6%) of the 88 patients with hemorrhage eventually died, compared to 23 (6.5%) in the non-bleeding group. This significant difference in mortality rates indicates that hemorrhage leads to a poor prognosis, indirectly confirming that the severity of pancreatitis correlates with an increased likelihood of hemorrhage.

In recent years, with the popularization of minimally invasive step-up strategies, bleeding complications related to necrotic drainage and endoscopic debridement have increased, drawing more attention to such complications [22, 23]. Our retrospective study analyzed the correlation between endoscopic debridement and the risk of hemorrhage, further exploring the relationship between the frequency and timing of debridement and bleeding. Previous studies have suggested that multiple necrosectomies and endoscopic debridement may increase the risk of hemorrhage [16, 24]. However, in our study, although the bleeding group underwent more frequent endoscopic debridement compared to the non-bleeding group (20.4% vs. 15%), the frequency of endoscopic debridement did not significantly affect the risk of bleeding (*P* = 0.477). Similarly, there was no significant difference in the timing of initial endoscopic debridement between the two groups (*P* = 0.233). This finding is consistent with the results of a recent high-quality multicenter study [25], which found that most initial endoscopic debridement occurred after 30 days. We believe that the timing of endoscopic debridement is not an independent risk factor. This aligns with clinical practice, as early pancreatic necrosis encapsulation is immature, and intervening before this time can easily lead to bleeding. Regarding endoscopic debridement, although the bleeding group had a higher proportion of endoscopic debridement compared to the non-bleeding group (21.6% vs. 15.1%), the difference was not statistically significant (*P* = 0.138). Additionally, comparisons between the two groups in terms of different access routes, such as TEN and PEN, also showed no significant differences (*P* > 0.05). These findings are consistent with our center’s previous research comparing bleeding outcomes following different access routes for debridement [24]. The data from this study did not find that endoscopic debridement or multiple debridements increase the risk of hemorrhage, contrasting with previous beliefs that multiple debridements might elevate the risk of bleeding. Our findings suggest that endoscopic debridement and multiple debridements are not significantly associated with hemorrhage, likely due to the advancement and standardization of minimally invasive techniques. This conclusion is consistent with recent research findings from our center [26].

In this study, we explored the association between pancreatic necrosis, infection, and the risk of hemorrhage. The results showed a significant difference in pancreatic necrosis between the bleeding group and the non-bleeding group (*P* < 0.001). Notably, in the bleeding group, the larger the area of pancreatic necrosis, the higher the incidence of hemorrhage, suggesting a potential link between the extent of necrosis and increased hemorrhage risk (11.4% vs. 25.0% vs. 59.1%). However, both univariate and multivariate analyses did not identify the area of pancreatic necrosis as a risk factor for hemorrhage. This finding is consistent with the results of Gupta et al. [16] but differs from those of Elhence et al., who identified pancreatic necrosis, including extensive necrosis, as a risk factor for hemorrhage [27]. Although this study did not confirm a direct correlation between the area of pancreatic necrosis and hemorrhage, it indirectly suggests that patients with hemorrhage had larger areas of pancreatic necrosis and more severe conditions. It is important to note that this study primarily focused on SAP patients, which may have influenced the interpretation of the results. Previous domestic studies have found that IPN is associated with an increased risk of bleeding [28]. Likewise, international research has confirmed that IPN is more susceptible to bleeding complications [7]. In this retrospective study, the incidence of IPN in the hemorrhage group was significantly higher than in the non-hemorrhage group (80.7% vs. 18.2%, *P* < 0.001). Multivariate analysis showed that IPN is an independent risk factor for hemorrhage (OR: 4.407, 95% CI: 1.854–10.476, *P* < 0.001). Hemorrhagic events are more common in IPN, possibly because infected necrosis increases the likelihood of vascular injury. The degradation of local tissues and vessel walls induced by infection and bacterial metalloproteases in areas of infected necrosis may further elevate the risk of bleeding [21, 27].

Scoring systems reflect the severity of the condition, with higher scores indicating more severe disease and significant pancreatic structural damage. The disorganization of newly formed granulation tissue and blood vessels can increase the risk of hemorrhage [4, 6]. Previous studies have pointed out that the development of hemorrhage in SAP is related to the severity of the condition [5, 6]. In this study, the CTSI scores in the bleeding group were significantly higher than in the non-bleeding group (10 vs. 6, *P* < 0.001). Similarly, the proportion of patients with APACHE II scores ≥ 15 was higher in the hemorrhage group than in the non-bleeding group (80.7% vs. 14.8%, *P* < 0.001), indicating that the bleeding group had more severe conditions and poorer prognoses. Subsequent multivariate analysis showed that CTSI scores and APACHE II scores ≥ 15 are independent risk factors for hemorrhage in SAP patients, with odds ratios (OR) of 1.771 (95% CI: 1.434–2.187, *P* < 0.001) and 12.323 (95% CI: 5.015–30.282, *P* < 0.001), respectively. Studies investigating the risk factors for SAP combined with gastrointestinal bleeding have also identified APACHE II scores and CTSI scores as independent risk factors [13]. Additionally, related research has confirmed a significant correlation between CTSI scores and abdominal bleeding [12]. Similarly, a significant correlation between APACHE II scores and SAP combined with abdominal bleeding has been reported [21]. Therefore, elevated CTSI and APACHE II scores indicate that SAP patients are in a severe condition with an increased risk of bleeding, necessitating heightened attention from clinicians.

Reflecting on our previous study involving 145 cases of endoscopic debridement at our center, we noted that the risk of bleeding from endoscopic debridement was associated with renal failure, suggesting that renal failure might increase the risk of hemorrhage [24]. However, in our current study, while univariate logistic analysis identified renal failure and CRRT as influencing factors, multivariate logistic regression analysis did not consider renal failure an independent influencing factor. Instead, CRRT was identified as an independent influencing factor for hemorrhage (OR: 0.251, 95% CI: 0.094–0.670, *P* = 0.006). Interestingly, although CRRT might be perceived as a potential risk factor for bleeding, our combined predictive model showed a negative correlation between CRRT and hemorrhage (OR: 0.251), indicating that CRRT is a protective factor that reduces the risk of bleeding. When SAP is accompanied by renal dysfunction, patients often exhibit coagulation abnormalities [8]. Traditionally, bleeding is associated with coagulation dysfunction. In this study, there were significant differences in PT and APTT between the two groups. However, univariate and multivariate analyses revealed that coagulation abnormalities were not influencing factors for hemorrhage (*P* > 0.05). Considering that coagulation dysfunction is a common clinical feature of SAP, these factors might be confounded by the severity of the condition itself, as more severe pancreatitis is more likely to exhibit coagulation abnormalities. Some studies have suggested that thrombocytopenia significantly increases the risk of bleeding [29]. However, in this study, there was no significant difference in PLT between the two groups (*P* > 0.05). Our findings did not support this view; instead, the PLT values were higher in the bleeding group compared to the non-bleeding group (223 vs. 190). We believe this might be related to compensatory elevation of PLT due to bleeding. When SAP is accompanied by multiple organ dysfunction, the deposition of substances such as urea nitrogen, creatinine, and various inflammatory mediators on the walls of microvessels can cause endothelial damage and increase vascular permeability. Combined with coagulation dysfunction, this can lead to microcirculatory disturbances, thereby increasing the risk of hemorrhage in SAP patients [8]. The application of CRRT can effectively remove these substances that damage blood vessels, reduce inflammatory responses, and improve microcirculatory disturbances, thereby lowering the risk of bleeding. Additionally, in AP caused by hypertriglyceridemia, CRRT can rapidly reduce blood lipid levels and prevent further deterioration of the condition [30]. Therefore, CRRT not only can improve the condition of SAP but may also reduce the risk of hemorrhage.

ACS is one of the severe complications in SAP patients. Due to extensive inflammatory necrosis, edema, and significant exudation in the pancreas, intra-abdominal pressure increases. When this pressure remains above 20 mmHg and is accompanied by organ dysfunction, ACS may occur [15]. Previous studies have confirmed that ACS is associated with higher APACHE II scores and multiple organ dysfunction syndrome (MODS). MODS not only affects the respiratory and renal systems but also includes hemodynamic abnormalities, which, as the condition progresses, increase the risk of hemorrhage [31, 32]. In this study, the incidence of ACS was significantly higher in the bleeding group compared to the non-bleeding group (34.1% vs. 7.4%, *P* < 0.001). Multivariate analysis indicated that ACS is an independent risk factor for hemorrhage in SAP patients (OR: 3.685, 95% CI: 1.462–9.287, *P* = 0.006). Despite the limited literature, this finding underscores the importance of ACS in SAP patients, as it is significantly associated with poor prognosis [33]. Therefore, for SAP patients with ACS, appropriate decompression treatments such as PCD should be employed to reduce abdominal pressure and improve the condition [15]. The study analysis indicated that the bleeding group received PCD more frequently before bleeding compared to the non-bleeding group (52.3% vs. 25.9%, *P* < 0.001). However, multivariate analysis showed that PCD was not directly associated with bleeding, suggesting that PCD might be an effective decompression treatment without increasing the risk of hemorrhage [34]. Therefore, in managing PCD, it is essential to avoid iatrogenic vascular injury by selecting the appropriate timing, employing proficient operational techniques, and ensuring standardized management of postoperative drainage tubes. These measures are crucial in preventing bleeding.

Subgroup analysis results showed no significant differences between the two groups in baseline characteristics, complications, rebleeding rates, and mortality rates (*P* > 0.05). However, further comparison of pre-bleeding interventions and prognosis details revealed that the abdominal bleeding group had more endoscopic debridement interventions compared to the gastrointestinal bleeding group (23.6% vs. 18.2%). Additionally, the number of patients receiving PCD and requiring surgical interventions was significantly higher in the abdominal bleeding group (*P* < 0.05). This finding is consistent with a foreign study [16], suggesting that more surgical interventions are needed to control bleeding in cases of abdominal hemorrhage. The abdominal bleeding group had a significantly shorter interval to rebleeding compared to the gastrointestinal bleeding group (4 vs. 24, *P* = 0.031), indicating that abdominal bleeding is more likely to recur in a shorter time frame. SAP patients with abdominal bleeding often undergo more invasive interventions, reflecting greater destruction of the pancreatic structure. Additionally, the disorganization of newly formed granulation tissue and blood vessels may increase the risk of rebleeding.

The strength of this study lies in it being the largest retrospective analysis of SAP cases with hemorrhagic complications to date. However, we acknowledge several limitations: First, as a single-center retrospective study, there may be selection bias. Second, since not all bleeding cases were included, we might not have captured all clinically relevant factors and did not consider the impact of medications administered during treatment, which could influence our results. Third, although we used propensity score matching to balance baseline differences between the hemorrhage and non-hemorrhage groups and reduce confounding effects, we cannot entirely rule out the potential influence of all unmeasured or unconsidered factors [35]. Lastly, the findings of this study may not apply to all AP patients, as it only involved SAP patients and did not consider recurrent or mild AP.

In clinical practice, bleeding complications in SAP require significant attention from multidisciplinary teams. Early identification and assessment of high-risk factors for bleeding, along with enhanced awareness and diagnostic capabilities, are crucial for timely examinations and treatments. Implementing appropriate therapeutic strategies can improve treatment outcomes and reduce mortality rates. Therefore, future research should focus more on hemorrhagic complications in SAP, conducting multicenter and large-sample clinical studies to clarify the high-risk factors influencing SAP hemorrhagic complications. Future research directions may include further exploration of the mechanisms by which CRRT prevents SAP-related hemorrhage and in-depth studies on the different clinical characteristics and treatment responses of patients with abdominal bleeding versus gastrointestinal bleeding. Additionally, other potential influencing factors and interventions also warrant more comprehensive investigation.

## Conclusions

SAP combined with hemorrhagic complications indicates a poor prognosis, leading to prolonged hospital stays and increased mortality rates. This study focused on analyzing the influencing factors of hemorrhagic complications in SAP and identified CTSI scores, IPN, APACHE II scores ≥ 15, and ACS as independent risk factors for bleeding. Additionally, it was found that CRRT treatment might be a protective factor that reduces the risk of bleeding.

## Supplementary Information

Supplementary Material 1.

## Data Availability

All data generated or analyzed during this study are included in this published article.

## Abbreviations

AP: Acute pancreatitis
SAP: Severe acute pancreatitis
CTSI: Computed tomography severity index
IPN: Infected pancreatic necrosis
APACHE II: Acute physiology and chronic health evaluation II
ACS: Abdominal compartment syndrome
CRRT: Continuous renal replacement therapy
BMI: Body mass index
WBC: White blood count
PT: Partial time
APTT: Activated partial thromboplastin time
ALB: Albumin
Cr: Creatinine
CRP: C-reactive protein
PCD: percutaneous catheter drainage
EN: Endoscopic necrosectomy
TEN: Transluminal endoscopic necrosectomy
PEN: Percutaneous endoscopic necrosectomy
ERCP: Endoscopic retrograde cholangiopancreatography
PSM: Propensity score matching
OR: Odds ratio
CI: Confidence interval
IQR: Interquartile range
