# Supplementary material for: Analysis of factors influencing the complications of severe acute pancreatitis combined with hemorrhage
Source: BMC Gastroenterol. 2026 Jul 27;26:693. doi: 10.1186/s12876-026-05154-0 (PMC13692932; doi:10.1186/s12876-026-05154-0)
Supplement: Supplementary file 1 [file 12876_2026_5154_MOESM1_ESM.docx]

STROBE Statement—checklist of items that should be included in reports of observational studies

|  | Item No. | Recommendation | Page  No. | Relevant text from manuscript |
| --- | --- | --- | --- | --- |
| **Title and abstract** | 1 | (*a*) Indicate the study’s design with a commonly used term in the title or the abstract | 1 | The study design is indicated in the Abstract (Methods): "This study retrospectively analyzed clinical data from SAP patients admitted to our hospital between January 2019 and December 2022." |
|  |  | (*b*) Provide in the abstract an informative and balanced summary of what was done and what was found | 1–2 | The structured abstract (Background, Methods, Results, Conclusion) gives a balanced summary of what was done and found, e.g.: "Using propensity score matching and multivariate logistic regression analysis, we identified independent factors influencing hemorrhage… A nomogram prediction model was constructed, achieving an area under the ROC curve (AUC) of 0.924." |
| Introduction | | | |  |
| Background/rationale | 2 | Explain the scientific background and rationale for the investigation being reported | 2–3 | "Acute pancreatitis (AP) is a prevalent acute disease of the digestive system, with an increasing incidence in recent years… Recent high-quality review studies indicate that in-hospital mortality of SAP is associated with bleeding. However, the factors influencing the incidence of hemorrhagic complications in SAP are yet to be clarified." |
| Objectives | 3 | State specific objectives, including any prespecified hypotheses | 3 | "Therefore, we conducted a retrospective analysis of clinical data from SAP patients with bleeding complications admitted to our hospital. We aimed to identify the risk factors influencing SAP with bleeding complications, compare the differences between gastrointestinal and abdominal bleeding, and observe related prognoses." |
| Methods | | | |  |
| Study design | 4 | Present key elements of study design early in the paper | 3 | "We retrospectively included clinical data of SAP patients hospitalized in the Department of Gastroenterology at the 1st affiliated Hospital, Jiangxi Medical College, Nanchang University, from January 2019 to December 2022." |
| Setting | 5 | Describe the setting, locations, and relevant dates, including periods of recruitment, exposure, follow-up, and data collection | 3 | Setting and dates: "…SAP patients hospitalized in the Department of Gastroenterology at the 1st affiliated Hospital, Jiangxi Medical College, Nanchang University, from January 2019 to December 2022. The data were obtained from the database maintained by the Department of Gastroenterology at the First Affiliated Hospital, which contains comprehensive information on patients diagnosed with SAP." |
| Participants | 6 | (*a*) *Cohort study*—Give the eligibility criteria, and the sources and methods of selection of participants. Describe methods of follow-up  *Case-control study*—Give the eligibility criteria, and the sources and methods of case ascertainment and control selection. Give the rationale for the choice of cases and controls  *Cross-sectional study*—Give the eligibility criteria, and the sources and methods of selection of participants | 3–4 | Participants were identified from the departmental SAP database. "Inclusion criteria: Complies with SAP diagnostics. Exclusion criteria: (1) Patients with SAP due to post-ERCP procedures, trauma, pregnancy, and chronic pancreatitis (n=23); (2) Patients who experienced bleeding or underwent surgery and debridement before admission (n=22); (3) patients with concurrent severe underlying conditions affecting the heart, lungs, liver, and kidneys (n=20); (4) cases with missing data (n=17); (5) patients with concurrent gastrointestinal and abdominal bleeding (n=8)." In-hospital outcomes (mortality, length of stay) were ascertained from medical records during hospitalization. |
|  |  | (*b*) *Cohort study*—For matched studies, give matching criteria and number of exposed and unexposed  *Case-control study*—For matched studies, give matching criteria and the number of controls per case | 6–7 | "We performed 1:4 nearest neighbor matching without replacement using a caliper value of 0.1 in the propensity score matching (PSM)… This resulted in 440 successfully matched SAP patients, including 88 patients in the bleeding group and 352 patients in the non-bleeding group." Matching variables: gender, age, BMI, smoking and drinking habits, diabetes, hypertension, and etiology. |
| Variables | 7 | Clearly define all outcomes, exposures, predictors, potential confounders, and effect modifiers. Give diagnostic criteria, if applicable | 4-5 | Outcome (hemorrhage) defined: "Gastrointestinal bleeding is defined as active bleeding from hematemesis, melena, or nasogastric tube bleeding…"; "Abdominal bleeding is defined as active bleeding characterized by blood drainage through a drain… or enhanced CT imaging showing high-density areas consistent with abdominal bleeding." Exposures/potential confounders (CTSI score, APACHE II score, IPN, organ failure, sepsis, ACS, PCD, EN, ERCP, mechanical ventilation, CRRT, laboratory indices, etc.) are listed under "Observational Indicators"; SAP, organ failure (modified Marshall score), sepsis, IPN and ACS are defined according to the cited diagnostic criteria. |
| Data sources/ measurement | 8* | For each variable of interest, give sources of data and details of methods of assessment (measurement). Describe comparability of assessment methods if there is more than one group | 4–5 | "All pertinent data pertaining to laboratory tests, imaging, scoring systems, complications, and interventions recorded within the 3 days preceding the hemorrhagic event were collected… these data can be considered pre-bleeding-related data, providing a more accurate reflection of the patient's condition before the onset of bleeding." All data were obtained from the same institutional database, so assessment methods were comparable between the bleeding and non-bleeding groups. |
| Bias | 9 | Describe any efforts to address potential sources of bias | 6–7 | "We applied the propensity score matching method to effectively balance the baseline differences between the bleeding and non-bleeding groups, thereby reducing the influence of confounding variables and enhancing the reliability of the results." Cases with missing data were excluded (n=17, p.4). |
| Study size | 10 | Explain how the study size was arrived at | 6-7 | All consecutive eligible SAP patients admitted during the study period were included: "This study retrospectively analyzed the clinical data of 894 SAP patients hospitalized in our department from January 2019 to December 2022. After applying the inclusion criteria, 90 cases were excluded, leaving 804 SAP patients in the study." No a priori sample-size calculation was performed. |

Continued on next page

| Quantitative variables | 11 | Explain how quantitative variables were handled in the analyses. If applicable, describe which groupings were chosen and why | 6–7 | "For normally distributed quantitative data, we described it by mean ± standard deviation; for non-normally distributed quantitative data, we used the median and interquartile range (IQR)." For regression analyses the APACHE II score was categorized as ≥15 vs <15. |
| --- | --- | --- | --- | --- |
| Statistical methods | 12 | (*a*) Describe all statistical methods, including those used to control for confounding | 6–7 | "The t-test and Mann–Whitney U test was used for quantitative data comparison… the Chi-square test and Fisher's exact probability method [for qualitative data]. Variables with significant differences between the two groups were included in univariate and multivariate logistic regression analysis (backward method) to identify independent influencing factors of SAP-associated hemorrhage. Results were expressed as odds ratio (OR) with a 95% confidence interval (CI)." Confounding was controlled by propensity score matching and multivariate logistic regression. A nomogram was constructed with the rms package in R 4.3.2; the ROC curve and AUC were calculated; analyses used SPSS 26.0, with P<0.05 considered significant. |
|  |  | (*b*) Describe any methods used to examine subgroups and interactions | 9-10 | Subgroup analysis by bleeding site: "To further investigate the differences between gastrointestinal bleeding and abdominal bleeding, we conducted a detailed subgroup analysis (see Tables 5 and 6)." |
|  |  | (*c*) Explain how missing data were addressed | 4 | "Cases with missing data (n=17)" were excluded (exclusion criterion 4); a complete-case analysis was therefore performed. |
|  |  | (*d*) *Cohort study*—If applicable, explain how loss to follow-up was addressed  *Case-control study*—If applicable, explain how matching of cases and controls was addressed  *Cross-sectional study*—If applicable, describe analytical methods taking account of sampling strategy | N/A | Not applicable — this was a retrospective study of hospitalized patients; in-hospital outcomes were obtained from medical records, so there was no loss to follow-up. Matching of the bleeding and non-bleeding groups was addressed by 1:4 propensity score matching (see item 6b). |
|  |  | (*e*) Describe any sensitivity analyses | N/A | No sensitivity analyses were performed. |
| Results | | | | |
| Participants | 13* | (a) Report numbers of individuals at each stage of study—eg numbers potentially eligible, examined for eligibility, confirmed eligible, included in the study, completing follow-up, and analysed | 6-7 | "This study retrospectively analyzed the clinical data of 894 SAP patients hospitalized in our department from January 2019 to December 2022. After applying the inclusion criteria, 90 cases were excluded, leaving 804 SAP patients in the study. Among these patients, 97 (12.1%) experienced bleeding complications…" After propensity score matching, 440 patients (88 bleeding, 352 non-bleeding) were analyzed. |
|  |  | (b) Give reasons for non-participation at each stage | 4–5 | Reasons for exclusion: SAP due to post-ERCP procedures, trauma, pregnancy, and chronic pancreatitis (n=23); bleeding or surgery and debridement before admission (n=22); concurrent severe underlying conditions of the heart, lungs, liver, and kidneys (n=20); missing data (n=17); concurrent gastrointestinal and abdominal bleeding (n=8). |
|  |  | (c) Consider use of a flow diagram | 6-7 | "The study flowchart is shown in Fig. 1." |
| Descriptive data | 14* | (a) Give characteristics of study participants (eg demographic, clinical, social) and information on exposures and potential confounders | 7-8 | "The baseline characteristics of the two patient groups are presented in Table 2, encompassing demographics, medical history, laboratory indicators, complications, imaging data, and clinical scoring systems." Characteristics before and after matching are shown in Table 1 (pp.6–7); pre-bleeding interventions and prognostic information are shown in Table 3 (pp.7–8). |
|  |  | (b) Indicate number of participants with missing data for each variable of interest | 4 | Cases with missing data were excluded (n=17; exclusion criterion 4); analyses were based on complete cases, so there were no missing data for the variables analyzed. |
|  |  | (c) *Cohort study*—Summarise follow-up time (eg, average and total amount) | N/A | Not applicable — there was no post-discharge follow-up; outcomes were assessed during hospitalization (ICU stay, total hospital stay, in-hospital mortality). |
| Outcome data | 15* | *Cohort study*—Report numbers of outcome events or summary measures over time | 6–8 | "Among these patients, 97 (12.1%) experienced bleeding complications, with 36 cases (37.1%) of gastrointestinal bleeding and 61 cases (62.9%) of abdominal cavity bleeding." In-hospital mortality: 46.6% (41/88) in the bleeding group vs 6.5% (23/352) in the non-bleeding group (P<0.001). |
|  |  | *Case-control study—*Report numbers in each exposure category, or summary measures of exposure | N/A | Not applicable — this is not a case-control study. |
|  |  | *Cross-sectional study—*Report numbers of outcome events or summary measures | N/A | Not applicable — this is not a cross-sectional study. |
| Main results | 16 | (*a*) Give unadjusted estimates and, if applicable, confounder-adjusted estimates and their precision (eg, 95% confidence interval). Make clear which confounders were adjusted for and why they were included | 8–9 | Unadjusted (univariate) and confounder-adjusted (multivariate) estimates are reported in Table 4: "CTSI score (OR: 1.771, 95% CI: 1.434–2.187, P<0.001), IPN (OR: 4.407, 95% CI: 1.854–10.476, P<0.001), APACHE II score≥15 (OR: 12.323, 95% CI: 5.015–30.282, P<0.001), ACS (OR: 3.685, 95% CI: 1.462–9.287, P=0.006), and CRRT (OR: 0.251, 95% CI: 0.094–0.670, P=0.006) were independent influencing factors of SAP-related bleeding." Variables that differed significantly between the two groups in Tables 2 and 3 were entered into the regression; confounding was further controlled by propensity score matching. |
|  |  | (*b*) Report category boundaries when continuous variables were categorized | 8–9 | The APACHE II score was categorized at a boundary of 15 (≥15 vs <15); other quantitative variables (e.g., CTSI score) were analyzed as continuous variables. |
|  |  | (*c*) If relevant, consider translating estimates of relative risk into absolute risk for a meaningful time period | 6–8 | Absolute risks are reported: bleeding occurred in 97 of 804 SAP patients (12.1%); in-hospital mortality was 46.6% in the bleeding group vs 6.5% in the non-bleeding group (P<0.001). |

Continued on next page

| Other analyses | 17 | Report other analyses done—eg analyses of subgroups and interactions, and sensitivity analyses | 8-9 | "By including the five independent influencing factors, we drew the ROC curve… When the five influencing factors were combined into a single prediction model, the AUC for the combined prediction of bleeding risk was 0.924 (95% CI: 0.889–0.959)"; subgroup analysis of gastrointestinal vs abdominal bleeding (Tables 5 and 6). |
| --- | --- | --- | --- | --- |
| Discussion | | | | |
| Key results | 18 | Summarise key results with reference to study objectives | 10–11 | "In our study, out of 804 SAP cases, 97 (12.1%) developed hemorrhages, with a median bleeding time of 30 days… the mortality rate of patients with hemorrhage was significantly higher than that of non-bleeding patients (46.6% vs. 6.5%, P<0.001)." |
| Limitations | 19 | Discuss limitations of the study, taking into account sources of potential bias or imprecision. Discuss both direction and magnitude of any potential bias | 15-16 | "First, as a single-center retrospective study, there may be selection bias. Second, since not all bleeding cases were included, we might not have captured all clinically relevant factors and did not consider the impact of medications administered during treatment, which could influence our results. Third, although we used propensity score matching to balance baseline differences…, we cannot entirely rule out the potential influence of all unmeasured or unconsidered factors. Lastly, the findings of this study may not apply to all AP patients, as it only involved SAP patients and did not consider recurrent or mild AP." |
| Interpretation | 20 | Give a cautious overall interpretation of results considering objectives, limitations, multiplicity of analyses, results from similar studies, and other relevant evidence | 16 | "In clinical practice, bleeding complications in SAP require significant attention from multidisciplinary teams. Early identification and assessment of high-risk factors for bleeding, along with enhanced awareness and diagnostic capabilities, are crucial for timely examinations and treatments." Conclusions (p.16): "SAP combined with hemorrhagic complications indicates a poor prognosis… CTSI scores, IPN, APACHE II scores≥15, and ACS as independent risk factors for bleeding. Additionally, it was found that CRRT treatment might be a protective factor that reduces the risk of bleeding." |
| Generalisability | 21 | Discuss the generalisability (external validity) of the study results | 15-16 | "The findings of this study may not apply to all AP patients, as it only involved SAP patients and did not consider recurrent or mild AP." |
| Other information | |  | | |
| Funding | 22 | Give the source of funding and the role of the funders for the present study and, if applicable, for the original study on which the present article is based | 17-18 | "The study design and data collection were funded by the National Natural Science Foundation of China (No: 81760121, 82060125 and 82100686), Funding scheme for outstanding young talent in Jiangxi Province (20192BCB23021), Jiangxi Provincial Natural Science Foundation (20202BAB206010 and 20212BAB216015) and Youth Talent Project of the 1^st^ affiliated hospital, Jiangxi Medical College, Nanchang University (YFYPY202006)." |

*Give information separately for cases and controls in case-control studies and, if applicable, for exposed and unexposed groups in cohort and cross-sectional studies.

**Note:** An Explanation and Elaboration article discusses each checklist item and gives methodological background and published examples of transparent reporting. The STROBE checklist is best used in conjunction with this article (freely available on the Web sites of PLoS Medicine at http://www.plosmedicine.org/, Annals of Internal Medicine at http://www.annals.org/, and Epidemiology at http://www.epidem.com/). Information on the STROBE Initiative is available at www.strobe-statement.org.
